# Mechanical homeostasis in tissue equivalents: a review

**DOI:** 10.1007/s10237-021-01433-9

**Published:** 2021-03-08

**Authors:** Jonas F. Eichinger, Lea J. Haeusel, Daniel Paukner, Roland C. Aydin, Jay D. Humphrey, Christian J. Cyron

**Affiliations:** 1 Institute for Computational Mechanics, Technical University of Munich, 85748 Munich, Germany; 2 Department of Biomedical Engineering, Yale University, New Haven, CT 06520 USA; 3 Institute of Continuum and Materials Mechanics, Hamburg University of Technology, 21073 Hamburg, Germany; 4 Institute of Material Systems Modeling, Helmholtz-Zentrum Geesthacht, 21502 Geesthacht, Germany

**Keywords:** Mechanical homeostasis, Tensional homeostasis, Mechanobiology, Mechanoregulation, Mechanotransduction, Mechanosensation, Growth and remodeling

## Abstract

There is substantial evidence that growth and remodeling of load bearing soft biological tissues is to a large extent controlled by mechanical factors. Mechanical homeostasis, which describes the natural tendency of such tissues to establish, maintain, or restore a preferred mechanical state, is thought to be one mechanism by which such control is achieved across multiple scales. Yet, many questions remain regarding what promotes or prevents homeostasis. Tissue equivalents, such as collagen gels seeded with living cells, have become an important tool to address these open questions under well-defined, though limited, conditions. This article briefly reviews the current state of research in this area. It summarizes, categorizes, and compares experimental observations from the literature that focus on the development of tension in tissue equivalents. It focuses primarily on uniaxial and biaxial experimental studies, which are well-suited for quantifying interactions between mechanics and biology. The article concludes with a brief discussion of key questions for future research in this field.

**Supplementary Information:**

The online version contains supplementary material available at 10.1007/s10237-021-01433-9.

## Introduction

The important role of mechanics in governing biological form and function has been known since the time of Galileo Galilei (1564–1641) and Giovanni Borelli (1608–1667), that is, for at least four centuries (Cyron and Humphrey 2017; Piolanti et al. 2018). Yet, it was only about a century ago that Henry Gassett Davis and Julius Wolff succeeded in condensing this general notion into two precise statements, Davis’ law in 1867 and Wolff’s law in 1892, which established relations between mechanical loading and growth and remodeling (G&R) in soft and hard tissues, respectively (Davis 1867; Wolff 1892). Building on the ideas of Claude Bernard in 1865 (Bernard 1865), Walter Cannon introduced the central concept of homeostasis in the 1920s (Cannon 1929), as summarized in the influential book “The Wisdom of the Body” in 1932 (Cannon 1932), which introduces the concept of homeostasis as “coordinated physiological reactions which maintain most of the steady states in the body”. These laws and concepts can be considered as landmarks paving the way to modern mechanobiology by exposing the fundamental importance of mechanically regulated processes for tissue health and disease.

Due to rapidly improving experimental and computational techniques and technologies, substantial progress has been made in mechanobiology, especially since the mid-1970s. A corner stone of nearly all approaches is the hypothesis that there exists a preferred mechanical state—often referred to as homeostatic—toward which mechanobiological activity is targeted. This generally accepted assumption goes back at least to the mid-1960s and the seminal study of Wolinsky and Glagov (1967), which showed that the aorta in various mammals develops such that the tension per medial lamellar unit is nearly the same $(\sim 2$ N/m), implying a “target” value of medial stress on the order of $10^2$ kPa. Twenty years later, Bissel and Aggeler (1987) introduced the concept of dynamic reciprocity, presenting the idea of dynamic feedback loops between cells and the surrounding extracellular matrix (ECM) that ensure that this target state is maintained within a certain tolerance. Since then, changes in the mechanical properties of the ECM have been shown to effect crucial cellular processes such as migration (Grinnell and Petroll 2010; Hall et al. 2016; Xie et al. 2017; Kim et al. 2019), differentiation (Chiquet et al. 2009; Mammoto et al. 2012; Zemel 2015), and even survival (Bates et al. 1995; Zhu et al. 2002; Schwartz 2002; Sukharev and Sachs 2012) (outside-in effect). Conversely, cells establish tissue form and function during development, maintain tissue integrity in health, and adapt tissue in response to perturbations such as injury or disease (Cox and Erler 2011; Lu et al. 2011; Ross et al. 2013; Bonnans et al. 2014; Humphrey et al. 2014a) (inside-out effect). In short, cells are equipped with the complementary processes of mechanosensation and mechanoregulation which are assumed to be crucially involved in the promotion of tissue health and proper functionality. Cells constantly perceive cues from their extracellular environment, transduce them into intracellular signals, and react, for example, by adapting their contractile state. Transmembrane receptors such as integrins physically connect the actin cytoskeleton inside the cell to fibers of the ECM and thus enable the transfer of mechanical loads (Jiang et al. 2006; Cavalcanti-Adam et al. 2007; Lerche et al. 2019).

Among the many different experimental approaches for studying the cell-matrix interactions that govern such biological form and function, tissue equivalents have emerged as particularly useful. Often formed as reconstituted collagen- or fibrin-based gels, cell-seeded tissue equivalents are typically meant to be simple in vitro model systems, not ex vivo tissue-engineered materials for implantation in regenerative medicine. Our focus is on the use of tissue equivalents for studying the underlying mechanobiology; we leave to others the review of tissue-engineered constructs, often beginning as cell-seeded polymeric scaffolds. We note, nonetheless, that some investigators use the term tissue-engineered to describe tissues fabricated for basic science studies, including via decellularization and subsequent cell-seeding, and that have revealed important mechanobiological effects, as, for example, that aberrant matrix can corrupt the behavior of otherwise normal cells (Sewanan et al. 2019).

In a seminal paper on tissue equivalents, Brown et al. (1998) wrote: “We would define tensional homeostasis as the control mechanism by which fibroblasts establish a tension within their extracellular collagenous matrix and maintain its level against opposing influences of external loading.” This definition reflected well their specific observations in fibroblast-seeded collagen gels under particular conditions and further highlighted the importance of homeostasis in mechanobiology. With an additional twenty-plus years of hindsight, Stamenović and Smith (2020) wrote, “we define tensional homeostasis as the ability to maintain a consistent level of tension, with a low variability around a set point, across multiple length scales.” This latter definition is closer in concept to that put forth by Cannon (1929, 1932) who introduced the word homeostasis, noting his careful choice of the prefix “homeo” (derived from the ancient Greek “homoios” meaning “similar”, thus allowing some variation) rather than “homo” (derived from the ancient Greek “homos” meaning the “same”, implying rigid constancy). Some accounts in the literature appear to misinterpret data within this framework by ignoring the possibility of maintaining a steady state within a range, perhaps in part because the allowable extent of such a range is not easily known. We emphasize, further, that it is not yet clear what the cells sense (force, extension, stiffness, compliance, etc.), though it is clear that some cells are more responsive to shearing loads (endothelial), some to tensile loads (fibroblasts), and some to compressive loads (chondrocytes). Hence, we suggest that “tensional homeostasis” is too narrow of a definition, with possible further ambiguities arising due to individual interpretations of what a tension is: a tensile force (units of N), an actual tension (units of N/m, as in surface tension), or a tensile stress (units of N/$\text {m}^2$). For these and other reasons, we prefer “mechanical homeostasis” as a more general term to encompass different responses by different cell types under different conditions and to acknowledge that we as a community continue to seek what cells sense and regulate. One can thus define mechanical homeostasis broadly as a ubiquitous mechanobiological process by which soft tissues seek to maintain key regulated variables within a range near a preferred value, often called a homeostatic target or set-point. Importantly, homeostatic targets or ranges adapt in some cases (Davies 2016) and additional terms, including allostasis and rheostasis, have been used in such cases in different fields, including those focusing on different types of stressors (e.g., emotional stress; McEwen and Wingfield 2010). Alternatively, homeostatic targets or ranges can be overridden in other cases, including chronic inflammation (Chovatiya and Medzhitov 2014). Indeed, it has been suggested that it is the very adaptivity of homeostatic targets that renders particular biological systems susceptible to override and thus to particular diseases (Kotas and Medzhitov 2015). Notwithstanding the important roles of the immune system in promoting or preventing mechanical homeostasis, particularly by macrophages (Wynn et al. 2013; Okabe and Medzhitov 2016), we focus herein on mechanobiological responses over modest ranges from normal by cells such as fibroblasts, which have primary responsibility for establishing, maintaining, remodeling, and repairing extracellular matrix (Tomasek et al. 2002).

**Fig. 1:**
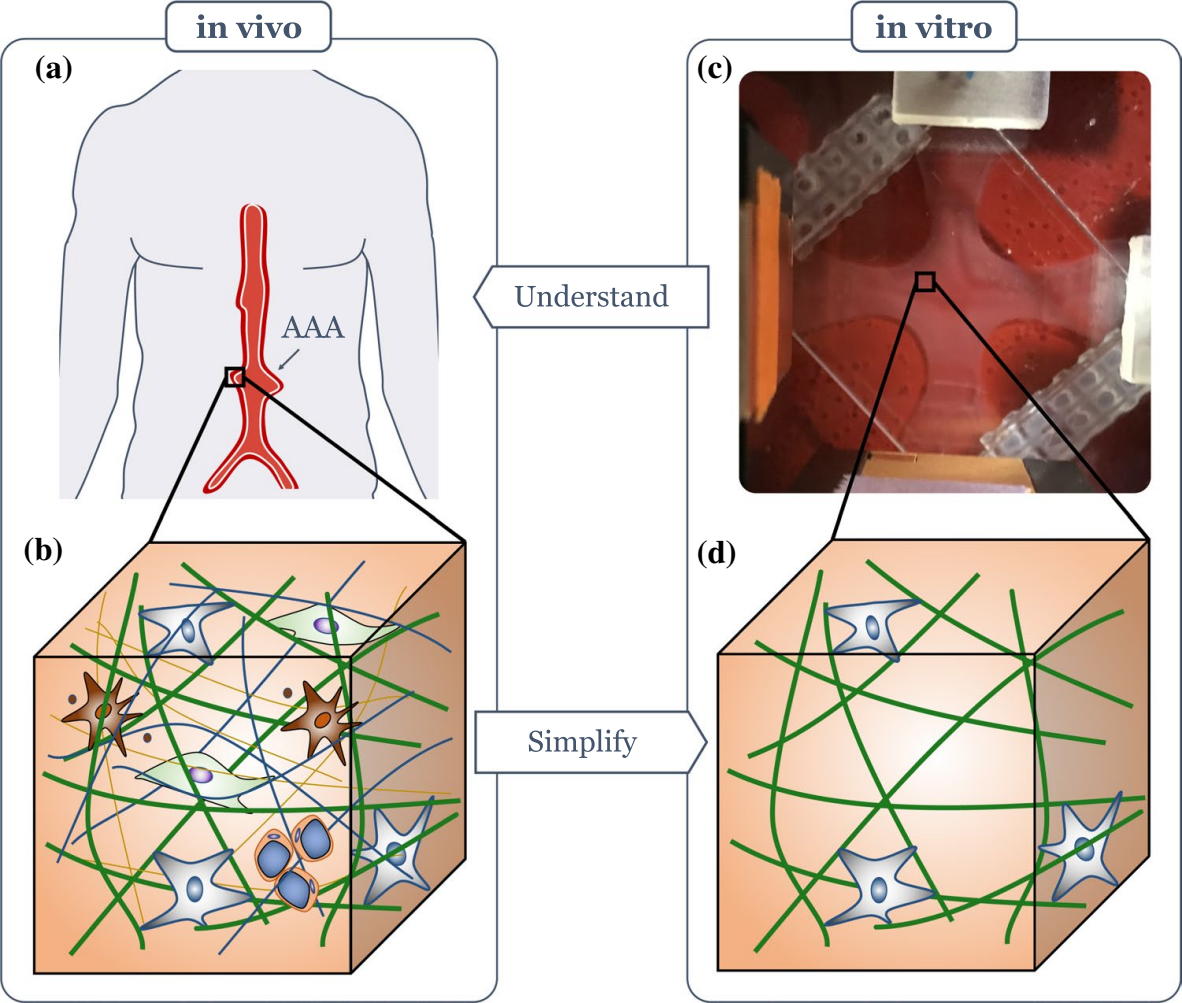
Cell-seeded collagen gels as tissue equivalents can provide information relevant to mechanical homeostasis of soft tissues. **a** Illustration of a patient with a local dilatation of the aorta (i.e., an aneurysm); due to ill-controlled mechanobiological processes; such aneurysms can continue to grow over years and often finally rupture, resulting in high mortality and morbidity; **b** in vivo studies often cannot provide the fine control needed to assess the cell-ECM interactions that are fundamental to promoting or preventing homeostasis, and thus understanding disease progression. **c** Cell-seeded collagen or fibrin gels have proven to be simple but powerful model systems to study soft tissue mechanobiology. **d** The much lower complexity of cell-seeded collagen gels compared to native ECM makes them useful for studying the fundamental cell-matrix interactions, often one cell type at a time

Awareness of the importance of mechanobiology and in particular mechanical homeostasis continues to grow in medicine and biomedical engineering. Some of the most important causes of mortality and morbidity in industrialized countries are closely linked to mechanobiology. These include diverse cardiovascular diseases and cancer (Weaver et al. 1997; Boettiger et al. 2005; Yeung et al. 2005; Weninger et al. 2009; Butcher et al. 2009; Lu et al. 2012). For example, thoracic aortic aneurysms appear to arise in part from dysfunctional mechanosensing or mechanoregulation of the ECM (Humphrey et al. 2014b), whereas abdominal aortic aneurysms (AAAs) (Fig. 1a, b) may experience continued enlargement due to mechanobiological instabilities (Cyron and Humphrey 2014; Cyron et al. 2014). While advances in medical imaging have increased the number of diagnosed aneurysms, a comprehensive understanding of their initiation and natural history, ranging from the sub-cellular to the organ scale, still remains elusive. There exists, therefore, a considerable opportunity to use allied methods, including in vitro studies of tissue equivalents (Fig. 1). Model systems—typically cell-seeded fibrin (Sander et al. 2011) or collagen (Sander and Barocas 2008) gels—are much simpler and therefore suitable for precise quantitative in vitro studies of cell-matrix interactions due to a greatly reduced number of confounding factors and interdependencies (Fig. 1c, d).

In this article, we review the current understanding of mechanical homeostasis in three-dimensional, gel-based tissue equivalents by summarizing and comparing results gained in different types of tissue culture experiments. Finally, we discuss limitations of the available data, raise open questions, and consider future directions of this emerging field of research.

## Using tissue equivalents to study mechanical homeostasis in soft tissues

**Fig. 2:**
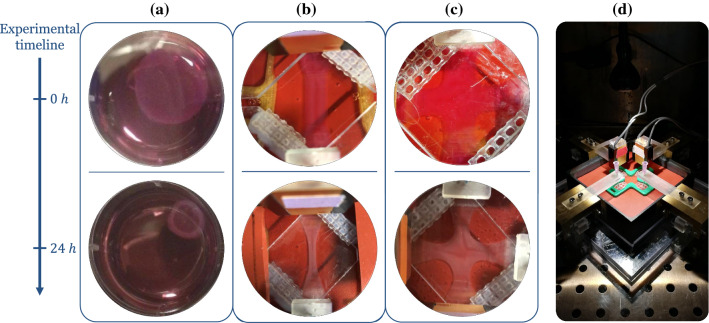
Free-floating (**a**), uniaxially constrained (**b**), and biaxially constrained (**c**) fibroblast-seeded collagen gels (i.e., tissue equivalents) are observed to contract substantially over the first hours to days. Cells residing in the gel initially spread and attach to surrounding collagen fibers. Cellular contraction then compacts the surrounding matrix thus stressing the fibers. **d** Biaxial testing device reported in Eichinger et al. (2020). Sensors are installed along two axes to record the development of tension in a cruciform gel

Mechanobiology focuses on understanding effects of mechanical stimuli on particular cellular actions. To study this, both the stimulus and the response need to be measurable and controllable; in other words, it is best to design experiments representing simple boundary and initial value problems that simplify data analysis and interpretation. Toward this end, in vitro studies using planar (rectangular or annular) or cylindrical tissue equivalents have been preferred. It is worth highlighting that cells in three-dimensional in vitro environments such as collagen or fibrin gels behave differently compared to those exposed to two-dimensional in vitro environments. The latter include micro-patterned substrates such as petri dishes made of plastic or glass coated with collagen or fibronectin. While such two-dimensional setups are simple, they cannot adequately mimic several important factors of the physiological environment in vivo. In fact, the dimensionality of the substrate interacting with cells has been shown to crucially influence cellular processes such as cell proliferation, survival, differentiation, and migration; see Baker and Chen (2012), Friedl et al. (2012), and Bonnier et al. (2015). For a review of two-dimensional versus three-dimensional culture environments, see Baker and Chen (2012) and Duval et al. (2017). The studies with three-dimensional tissue equivalents can be classified further by the mechanical boundary conditions imposed experimentally. So far, free-floating (mostly circular), uniaxially constrained or extended, and biaxially constrained or extended cell-seeded gels have been used in most experiments (Fig. 2). Cylindrical tissue equivalents that generate hoop stresses and circumferential fiber alignment if set around a non-adhesive mandrel have been used similarly (Barocas et al. 1998; Isenberg et al. 2006), but are less common and are not discussed in detail here. In most cases, cylindrical geometries are motivated by tissue engineering applications, including vascular grafts.

### Circular free-floating tissue equivalents

First, free-floating collagen discs were introduced by Bell et al. (1979), who showed that the gels compact significantly ($\sim 90\%$ Fig. 2a) due to cellular mechanobiological activity over a period of days. Free-floating discs are experimentally simple to create and handle and thus are favorable for qualitative studies (Steinberg et al. 1980; Buttle and Ehrlich 1983; Grinnell and Lamke 1984; Ehrlich et al. 1986; Woodley et al. 1991; Schiro et al. 1991; Kelynack et al. 2000; Ehrlich and Rittenberg 2000; Grinnell 2000; Grinnell and Ho 2002; Redden and Doolin 2003; Orlandi et al. 2005; Stevenson et al. 2010; Grinnell and Petroll 2010). Nevertheless, due to the relatively complex residual stress fields that develop (Simon et al. 2012, 2014), it can be challenging to determine key parameters or mechanisms of soft tissue mechanobiology quantitatively from experiments with free-floating discs. For example, as mentioned before, one key question is how mechanical stimuli are transduced and then drive cellular actions via corresponding signaling pathways. To obtain a quantitative understanding of this problem, experiments are required where both the mechanical loads and the subsequent cellular responses can be quantified accurately. Given that prior studies using free-floating gels have not interpreted regional cellular responses in terms of the complex inhomogenous stress field (compressive and tensile stresses, which induce regional anisotropies), this review focuses instead on uniaxial and biaxial testing studies, which were introduced several decades after the first free-floating gels were studied, in part, to overcome limitations of the latter. For a review of free-floating collagen gels, see Dallon and Ehrlich (2008) and Simon and Humphrey (2014). Here, therefore, we turn our attention to uniaxial and biaxial studies wherein stress/strain fields can be homogeneous if studied sufficiently far from the end-effects at the boundaries.

### Uniaxially constrained tissue equivalents

**Fig. 3:**
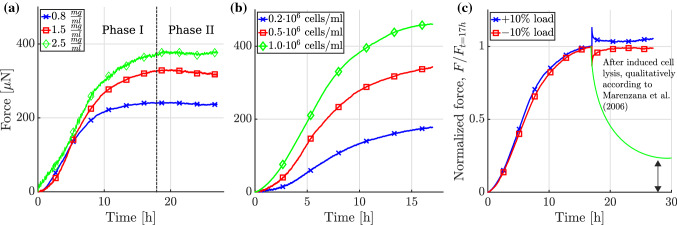
In uniaxially constrained tissue equivalents, the measured tensile force evolves in two characteristic phases: a steep increase (phase I) followed by a plateau (phase II). Here, we show experimental data from Eichinger et al. (2020). Each curve is an average of three identical experiments. **a** Dependence of the plateau value of tension on collagen concentration in the gel with a constant cell density of $0.5 \cdot 10^6 \text {cells/ml}$. Higher collagen concentrations lead to higher steady state values. **b** Influence of cell density for a collagen concentration of 1.5 mg/ml : Higher cell densities lead to both a steeper initial increase and a higher plateau value of the measured force. **c** If the tissue equivalent (collagen concentration 1.5 mg/ml , cell density $1.0 \cdot 10^6$ cells/ml) is suddenly stretched or released after having reached the plateau state, force returns toward its value prior to this perturbation. The monotonically decreasing force qualitatively illustrates the expected evolution in case of cell lysis according to Marenzana et al. (2006); the fraction of the tensile force remaining in the gel has been called residual matrix tension (RMT)

**General setup** For quantitative studies of how cells respond to external mechanical loading, uniaxially constrained or extended tissue equivalents (Fig. 2b) were developed. Unlike free-floating gels, uniaxial setups exhibit a largely homogeneous tension field (within the central region), with measurements of the net uniaxial loading straightforward. This makes it easier to quantify how a certain mechanical target state evolves over time, and if and how it is restored after perturbations. Most uniaxial experiments with tissue equivalents follow a similar approach. First, a gel is fabricated by combining collagen, cells, Dulbecco’s modified Eagle’s medium (DMEM), a buffer, and an antibiotic (for more details, see supplementary Tables S1 and S2). The collagen solution is then cast into a rectangular mold within a device where it sets around two insets, one of which can be connected to a motor that can impose uniaxial strain. The other inset, residing on the opposite side of the gel, is connected to a force transducer such that forces imposed on or generated within the gel can be measured. Typically, the gel is initially free of mechanical load. If, however, the total length of the gel is kept constant by the device, one observes that a substantial tension builds up over a period of several hours due to cell-mediated contraction of the gel (phase I), often reaching a steady state (phase II) (Fig. 3). This steady state is often referred to as a homeostatic state—thus reflecting Brown’s tensional homeostasis. As the tissue evolves, the initial rectangular shape of the gel typically changes to a more dog-bone like shape because cell-mediated contraction acts not only along the gel axis, but also transversely, where it is not impeded as the lateral surface remains traction-free (Fig. 2b). Studies with tissue equivalents in such a uniaxial setting have addressed in particular the following aspects so far.

**Mechanical homeostasis** In contrast to classical engineering materials, living soft tissues apparently seek to establish and maintain a preferred mechanical state that is not stress-free. Delvoye et al. (1991) were the first to show that uniaxially constrained collagen gels seeded with dermal fibroblasts exhibit a characteristic behavior with the aforementioned two distinct stages. During the first $6-12 \ \text{h}$, a tensile force increases rapidly (phase I) before entering a steady state (’homeostatic state’) with neither further increase nor decrease (phase II). When this tension is perturbed by slightly stretching or releasing a gel that has already reached phase II, tension returns toward the prior level within around $1\ \text{h}$. To date, it remains controversial within what tolerance the prior level is restored, mainly or only to some extent, but we must recall here Cannon’s particular choice of the prefix homeo, meaning "similar", not "the same". The two stages observed by Delvoye et al. (1991) were confirmed by Eastwood et al. (1994, 1996), who found that even cell-free constrained collagen gels tend to build up some tension during the first few hours of maturation, presumably due to entanglement and cross-linking of fibers in the gel. As noted earlier, it was a few years later that this group coined the terminology "tensional homeostasis" to describe their observations in these uniaxially constrained, fibroblast-populated gels under particular fabrication and culture conditions. Namely, they examined cell-populated collagen-glycosaminoglycan sponges subjected to different loading protocols, including cyclical over- and under-loading, cyclical median loading, and cyclical incremental loading, to study reactions of the gels triggered by these diverse external stimuli. When gels were subjected to a sudden stretch or release, gels were observed to return toward their prior tension level in the following 15 min. Especially notable is that the tension in the gels was observed to re-increase immediately after release, which led the authors to the conclusion that viscoelastic effects alone are not sufficient to explain the observed behavior.

The two characteristic stages of this so-called tensional homeostasis and the tendency of tissue equivalents to restore their prior tension level after perturbation were confirmed by Ezra et al. (2010). They observed the homeostatic state to arise during the first 8 h. When this state was perturbed by a series of alternate stretch and release steps every 30 min, tension was observed to return toward the prior value during the 30 min between two consecutive perturbation steps. However, the 30 min relaxation periods between two subsequent perturbations were too short to answer the question within which tolerance the homeostatic state is restored after a perturbation. Generally, Ezra et al. (2010) found that gels reacted more strongly to perturbations if their amplitude was higher. Very similar results were reported by Bisson et al. (2009), where gels were observed to counteract uniaxially applied loads. In cases of reduced loading, gels reacted by increasing tension; in cases of increased loading, gels reacted by decreasing tension. Furthermore, it was observed that fibroblasts from diseased fibrotic tissue did not replicate the aforementioned characteristic two-stage behavior—steep increase of tissue tension (phase I) followed by a plateau (phase II), because they failed to reach the second stage characterized by steady state at least within the same time interval.

The observations summarized above were further supported by Marenzana et al. (2006), who studied the effect of cytochalasin D. This agent disrupts the actin cytoskeleton and thereby eliminates active cellular contraction from the gel. When added to the culture medium after 24 h, when the homeostatic state had been reached, it induced a rapid and total decline of the tension in gels populated with human dermal fibroblasts. No residual matrix tension (RMT) was measured in this case. Interestingly, in gels populated with fibroblasts from rat tendon, a substantial residual tension was observed even 12 h after treatment with cytochalasin D. Hypo-osmotic cell lysis via replacement of the culture medium with distilled water gave similar results regarding the measurable RMT. When comparing the results of adding cytochalasin D after 4 h and after 60 h in culture, RMT seemed to increase linearly with time in culture, making up a growing fraction of the tension before treatment. RMT was also higher when gels had been exposed to cyclical loading. Moreover, it was shown that cell-seeded collagen gels after 24 h had a significantly higher stiffness than cell-free gels after the same time in culture. Collectively, these observations suggest that at least certain types of fibroblasts, such as the ones from rat tendon, remodel the matrix surrounding them in a way that is not purely elastic (Yamato et al. 1995; Sawhney and Howard 2002). Such cells rather appear to start by elastically contracting their surrounding gel; subsequently, they seem to entrench an increasing fraction of this tension, possibly because this is energetically more favorable than maintaining it over a prolonged time by active cellular tension. It remains an open question why cyclical loading increases RMT. It is noteworthy that Kolodney and Wysolmerski (1992) did not observe any RMT for chick embryo fibroblasts or endothelial cells, despite following an approach very similar to the one of Marenzana et al. (2006). This further supports the hypothesis that the entrenchment of matrix tension—perhaps via physical entanglements, but also via matrix cross-linking via transglutaminases (when remodeled) or lysyl oxidases (when synthesized) as noted before (Simon et al. 2014)—depends on the cell type and experimental conditions, including the type of matrix used to constitute the gel and the culture media (e.g., whether containing copper or not, noting that enzymes such as lysyl oxidase are copper dependent).

**Influence of cell density and matrix composition on tension** When using both calf skin fibroblasts (Delvoye et al. 1991) and NIH 3T3 fibroblasts (Eichinger et al. 2020), tension in the homeostatic state of uniaxial tissue equivalents increases linearly with collagen concentration. Notably, Eichinger et al. (2020) demonstrated that higher collagen concentrations increase the steady state tension but not the rate at which tension is built up initially. Moreover, tissue tension increases with cell density, either linearly (Delvoye et al. 1991; Eichinger et al. 2020) or nonlinearly (Legant et al. 2009; Jin et al. 2015) depending on the experimental conditions.

Increasing the collagen concentration also increases the gel stiffness (Alcaraz et al. 2011; Miroshnikova et al. 2011; Hall et al. 2016; Joshi et al. 2018). Therefore, one may hypothesize that the reason for the effect of the collagen concentration on the homeostatic tension is that cells often tend to respond more to a stiffer mechanical environment by increasing contraction (Ghibaudo et al. 2008; Califano and Reinhart-King 2010; Schiller and Fässler 2013; Cheng et al. 2013; Oria et al. 2014; Zhu et al. 2016). This hypothesis is supported by observations of Zhao (2014), who statically prestretched microtissues, which increased their stiffness, and subsequently observed higher cell-mediated tissue tension. It is interesting to note that the alignment of fibers and cells by static loading can reorganize the tissue, which can increase its anisotropic strength, stiffness, and ability to contract (Grenier et al. 2005). In general, mechanical homeostasis is promoted by the cells not only under static but also under dynamic loading (Walker et al. 2020), which can thus also be used to alter tissue stiffness.

It is noteworthy that Karamichos et al. (2007) reported that human dermal fibroblasts produced a much lower homeostatic tension and also a much slower development of the homeostatic tension plateau if exposed to an initially prestretched matrix. This effect was the more pronounced the higher the initial prestretch. Given that prestretch stiffens the collagen matrix, these observations may seem at first glance a contradiction to the ones of Delvoye et al. (1991), Zhao (2014), and Eichinger et al. (2020). Yet, both may be brought into agreement by noting that cells have been found to respond to changes in stiffness in a biphasic manner (Chan and Odde 2008), that is, there may exist a certain regime where cellular tension increases with the stiffness of the mechanical environment and another regime where the opposite is true. A similar phenomenon is also known for cell migration (Bangasser et al. 2017).

It has been shown that the effective stiffness that is sensed by the cells and that is determined by the matrix stiffness and the stiffness of the boundary (noting that stress and stiffness fields can often be computed away from the end effects, but cells near the boundaries yet sense and respond to their local environment) needs be considered for mechanoregulation (Kural and Billiar 2016). In particular, Legant et al. (2009) observed that both a higher gel stiffness and a stiffer fixation of the tissue at the boundary led to a higher cell-mediated tension in the tissue.

**Role of cell type and tissue origin** Multiple studies have shown that both the type of the cells used in tissue equivalents as well as the type of tissue from which they have been extracted affect the homeostatic tension. Delvoye et al. (1991) reported that calf dermal fibroblasts produced higher tension than human dermal fibroblasts. Active tension generated by chick embryo fibroblasts was found to be higher than that generated by a monolayer of human umbilical vein endothelial cells (Kolodney and Wysolmerski 1992). It is possible that the latter is not a consequence of the different cell type but rather because the endothelial cells were seeded on the surface of pre-polymerized gels, not embedded within the gel. On the other hand, it is also noteworthy that the endothelial cells reached their homeostatic state only after 4–5 days, whereas the fibroblasts did so in less than 24 h, which may indicate a more pronounced contractile activity of the fibroblasts. Interestingly, neither the chick embryo fibroblasts nor the endothelial cells entrenched any RMT, proven by a rapid decay of the tension to zero after addition of Cytochalasin D. By contrast, Marenzana et al. (2006) reported RMT for rat tendon fibroblasts as mentioned above, which suggests that generation of RMT may depend on factors not yet fully understood. Also Eastwood et al. (1996) demonstrated considerable dependency on the cell type and cell line for both the level of homeostatic tension and the rate at which it is built up initially. Rabbit tendon fibroblasts, for example, only started contracting after 14 h in culture and produced much less tension than human dermal fibroblasts. It was also found that there is a difference in homeostatic tension between human dermal fibroblasts from the same tissue sample depending on whether the fibroblasts were grown by explant migration or extracted by bacterial collagenase digestion. Fibroblasts grown from explants generated a tension approximately $60\%$ higher than the one generated by fibroblasts extracted with collagenase. This effect was attributed to a natural selection bias. The cells that migrated most strongly were favored by the explant growth extraction process so that this process selected a sub-population of cells that distinguished itself by unusually strong cellular contractility. Note that Ezra et al. (2010) studied mechanical homeostasis with occular fibroblasts from individuals with and without floppy eyelid syndrome. The latter is associated with a reduced stiffness of the tarsal plate and thus a supposedly higher (dynamic) tension in vivo. Interestingly, fibroblasts from individuals with floppy eyelid syndrome were also observed to establish a higher homoestatic tension when extracted from their natural environment and studied in vitro. A possible explanation for this is that cells may alter their homeostatic target state when exposed to altered mechanical or possibly biochemical conditions over a prolonged period. Such a possibility is consistent with a generalized concept of adaptive homeostasis (Davies 2016).

Porcine smooth muscle cells from different layers of the pulmonary artery were found to establish (within around one day) a homeostatic tension at different speeds and with different magnitudes. Both were higher for cells from the outer medial layer than for cells from the inner medial layer (Hall et al. 2007). The tension generated by fibroblasts from different regions of the human eye were studied by Dahlmann-Noor et al. (2007). Corneal fibroblasts established a much higher tension than fibroblasts from Tenon’s layer. The tension generated by scleral fibroblasts was barely measurable. Notably, fibroblasts from the same organ differ significantly in their ability to generate tension. The authors also determined the intrinsic cellular tension of each fibroblast type, which was defined as the plateau tension divided by the mean cell volume. When comparing intrinsic tension with the ones measured during the experiment, the intrinsic cellular tension was at least of a similar order of magnitude. Cell size could therefore be an important factor influencing the homeostatic plateau level of tension. It is worth mentioning that Bisson et al. (2004, 2009) observed for Dupuytren’s fibroblasts a significantly higher tension than for control fibroblasts but no plateau of tension. A likely explanation for the latter phenomenon is that for these cells the time to reach the homeostatic state was simply longer than the period for which the experiments were run. Another possible explanation would be the loss of the ability of these fibroblasts to control the mechanical state of their surrounding tissue. Interestingly, they also found that these cells reacted after the first of four consecutive uniaxial overloading events with a further increase in contraction, that is, not following the concept of homeostasis which would suggest a reduced contraction so that the gel tension returns toward the preferred plateau value. An uncontrolled contraction of fibroblasts in Dupuytren’s tissue, regardless of the mechanical stimulus, could explain why patients stretching their fingers to overcome the disease particularly suffer from severe disease. Finally, Sawadkar et al. (2020) found that tendon fibroblasts produced lower tension with an increasing passage number.

**Role of growth factors** As reported by Brown et al. (2002), TGF-$\beta$1 elevates both the rate and extent of cellular tension generation, although only up to a certain saturation value. TGF-$\beta$1 increases cell tension and this may trigger integrin expression and contractile protein expression, and thereby enable higher tissue tension (Ignotz and Massague 1986; Brown et al. 2002). Note that Bisson et al. (2009) demonstrated that both the early contraction rate after $2\ \text{h}$ and the rate after $20\ \text{h}$ of Dupuytren’s fibroblasts was significantly increased when stimulated with TGF-$\beta$1. Notably, TGF-$\beta$1 seems to affect also the cells’ response to external mechanical loads. If a tissue equivalent in its homeostatic state is stretched, mechanical homeostasis typically ensures a subsequent decay of the tension back toward the homeostatic level, which naturally requires a decrease in the contractile activity of the cells in the tissue equivalent. However, when the TGF-$\beta$1 treated cell-seeded gels were exposed in Bisson et al. (2009) to four consecutive uniaxial overloading events by rapidly increasing the length of the gel, a further increase was observed during the first three of these load steps. This suggests that the nodule and cord fibroblasts used in this experiment responded to the external stretch not by a decrease but rather by an increase of their contractile activity.

TGF-$\beta$1 also appears to affect inelastic matrix remodeling. TGF-$\beta$1 increases not only the overall tension generation by the cells, but also the mean RMT measurable after addition of Cytochalasin D (Marenzana et al. 2006). However, the ratio of RMT after addition of Cytochalasin D and the tension prior to the addition of Cytochalasin D was much smaller for TGF-$\beta$1 treated cells compared to cultures without TGF-$\beta$1, indicating that a lower percentage of tension is entrenched in the matrix.

Brown et al. (2002) also investigated effects of the concentration of fetal bovine serum (FBS) in the experimental culture medium. They found that in a medium with a concentration of $2\%$, the tension rose more slowly and to a lower homeostatic value compared to a medium with a concentration of $10\%$ FBS. The use of experimental medium without any FBS resulted in no tension generation. An increase in total tension generation for increasing concentrations of FBS was shown by Delvoye et al. (1991).

**Cell-matrix interactions** Cell-matrix interactions rely on the proper interplay of multiple components and substances. Kolodney and Wysolmerski (1992) studied the role of different parts of the cytoskeleton by treating the gels with drugs that selectively deactivated or disrupted particular constituents or interactions within the cytoskeleton. When Cytochalasin D—causing the disruption of actin filaments—was added to the culture medium after the plateau state had been reached, the entire tension disappeared rapidly for both chick embryo fibroblasts and human vein endothelial cells. These findings highlight that actin filaments are essential for mechanical homeostasis, both sensing and regulating the matrix. Conversely, treatment with the microtubule-disrupting drug nocodazole increased gel tension roughly by a factor of 2. Intact microtubules support cell shape and act against the tension of the actin cytoskeleton. Therefore, their disintegration may be expected to increase the contractile tension exerted by the cell on the surrounding tissue. Sethi et al. (2002) found on the basis of cell-populated glycosaminoglycan sponges that cells use their distinct receptor-ligand systems in a sequential order to exert tension on the surrounding matrix. Cells first attach through their fibronectin receptors, followed by their vitronectin receptors, and finally through their collagen receptors. It appears that if one of these stages is left out, cells can no longer generate the full tension through normal collagen attachment.

**Table 1:** Overview of experimental methods and results related to uniaxially constrained tissue equivalents seeded with human dermal fibroblasts (FB)

Cell type	Force $(\frac{\upmu N}{10^6 \, \mathrm{cells}})$	Cell density $(\frac{10^6 \, cells}{\text{ml}})$	Collagen concentration $\,(\frac{mg}{ml})$	Annotations	References
Human dermal FB	2522 (Max.)	0.20	0.46		Delvoye et al. (1991)
	2377 (Max.)	0.40			
Human dermal FB	126 (Plat.)	1.10	1.00		Eastwood et al. (1994)
Human dermal FB	260-609 (Max.)	Unknown	0.80	Different cell lines	Eastwood et al. (1996)
	333 (Plat.)			Extraction by collagenase digestion	
	516 (Plat.)			Extraction by explant growth	
Human dermal FB	355 (Max.)	1.00	1.50	No mAb 4B4 ($\beta$1-blocking antibody)	Jenkins et al. (1999)
	252 (Max.)			1 $\upmu$g/ml mAb 4B4	
	218 (Max.)			2 $\upmu$g/ml mAb 4B4	
Human dermal FB	$$\begin{array}{r} -54\ (\text{Plat.}) \\ 342\,(\text{Max.}) \\ 629\,(\text{Plat.}) \end{array}$$	1.00	1.70	$$\left. \begin{array}{l} 0\% \hbox { FBS}\\ 2\% \hbox { FBS}\\ 10\% \hbox { FBS}\end{array}\right\} \ \begin{array}{r} \text {Matched cell line and}\\ \text {Passage number }(*) \end{array}$$	Brown et al. (2002)
	$$\begin{array}{r} 334\,(\text{Max.}) \\ 254\ (\text{Max.}) \\ 544\,(\text{Max.}) \\ 515\,(\text{Max.}) \\ 82\,(\text{Max.}) \end{array}$$			$$\left. \begin{array}{l} 2\% \hbox { FBS, no TGF}-\beta 1\\ 2\% \hbox { FBS}, 2.5\, \hbox {ng/ml TGF}-\beta 1\\ 2\% \hbox { FBS}, 7.5\, \hbox {ng/ml TGF}-\beta 1\\ 2\% \hbox { FBS}, 15.0 \,\hbox {ng/ml TGF}-\beta 1\\ 2\% \hbox { FBS}, 30.0\, \hbox {ng/ml TGF}-\beta 1 \end{array}\right\} \ *$$	
	$$\begin{array}{l} 80\,(\text{Max.}) \\ 258\,(\text{Plat.}) \\ 381\,(\text{Max.}) \\ 118\,(\text{Max.}) \end{array}$$			$$\left. \begin{array}{l} 2\% \hbox { FBS, 2.5 ng/ml TGF}-\beta 3\\ 2\% \hbox { FBS}, 7.5 \,\hbox {ng/ml TGF}-\beta 3\\ 2\% \hbox { FBS}, 15.0 \,\hbox {ng/ml TGF}-\beta 3\\ 2\% \hbox { FBS}, \,30.0 \hbox {ng/ml TGF}-\beta 3 \end{array} \right\} \ *$$	
	$$\begin{array}{r} 199\,(\text{Max.}) \\ 623\,(\text{Plat.}) \end{array}$$			$$\left. \begin{array}{l} 2\% \hbox { FBS, no TGF}-\beta 1\ or -\beta 3\\ 2\% \hbox { FBS}, 12.5 \,\hbox {ng/ml TGF}-\beta 1 \\ 2\% \hbox {FBS},\ \ 15.0 \,\hbox {ng/ml TGF}-\beta 3 \end{array} \right\} \ *$$	
Human dermal FB	159 (Plat.)	0.29	1.83		Campbell et al. (2003)
	182 (Max.)				
Human dermal FB	129 (Plat.)	1.00	Unknown	0% prestrain, FBS addition after 0 h	Karamichos et al. (2007)
	17 (Max.)			5% prestrain, FBS addition after 0 h	
	34 (Max.)			10% prestrain, FBS addition after 0 h	
	179 (Plat.)			0% prestrain, FBS addition after 1 h	
	78 (Plat.)			5% prestrain, FBS addition after 1 h	
	15 (Plat.)			10% prestrain, FBS addition after 1 h	

Forces refer to experiments without (or before) any external strain applied. The culture medium contained 10% FBS unless indicated differently. Maximal forces (Max.) and plateau forces (Plat.) are normalized by the number of cells used in the experiment. Due to missing data, the possibly more suitable normalization (force/cross-sectional area)/(number of cells/gel volume) was not possible. Therefore, information about the dimensions of the tissue equivalents (cross-sectional area, length, volume) in future studies would facilitate the comparison between experiments

**Table 2:** Overview of experimental methods and results related to tissue equivalents seeded with cells other than human dermal fibroblasts (FB)

Cell type	Force $\,(\frac{\upmu \mathrm{N}}{10^6\,cells})$	Cell density $\,(\frac{10^6\,cells}{ml})$	Collagen concentration$\,(\frac{mg}{ml})$	Annotations	References
Human osteosarcoma FB	221 (Max.)	1.00	1.50	Without $\alpha$2$\beta$2 integrins	Jenkins et al. (1999)
	318 (Max.)			With $\alpha$2$\beta$2 integrins	
Human ocular FB	18 (Plat.)	7.00	1.50	Human scleral FB	Dahlmann-Noor et al. (2007)
	208 (Plat.)	1.00		FB human Tenon’s	
	311 (Plat.)	1.00		Human corneal FB	
Human tarsal plate FB	162 (Plat.)	1.00	1.58	Healthy upper eyelid FB	Ezra et al. (2010)
	437 (Plat.)			Floppy eyelid syndrome FB	
Human fascial tissue FB	80 (Max.)	1.00	1.88	Healthy carpal ligament (HCL)	Bisson et al. (2004)
	219 (Max.)			Dupuytren’s cord (DC)	
	290 (Max.)			Dupuytren’s nodule (DN)	
Human fascial tissue FB	91 (Max.)	1.00	1.88	HCL FB, without TGF-$\beta$1	Bisson et al. (2009)
	234 (Max.)			DC FB, without TGF-$\beta$1	
	262 (Max.)			DN FB, without TGF-$\beta$1	
	330 (Max.)			HCL FB, 2 ng/ml TGF-$\beta$1	
	428 (Max.)			DC FB, 2 ng/ml TGF-$\beta$1	
	560 (Max.)			DN FB, 2 ng/ml TGF-$\beta$1	
Calf dermal FB	5623 (Plat.)	0.20	0.46		Delvoye et al. (1991)
Chick embryo FB	585 (Plat.)	0.77	0.87		Kolodney and Wysolmerski (1992)
Rabbit tendon FB	124 (Max.)	Unknown	0.80	Endotenon FB	Eastwood et al. (1996)
	172 (Max.)			Sheath FB	
Rabbit tendon FB	168 (Plat.)	1.00	Unknown	P0, P = passage number	Sawadkar et al. (2020)
	126 (Max.)			P1	
	90 (Max.)			P3	
	51 (Max.)			P6	
Rat tendon FB	197 (Max.)	1.00	1.70	Without TGF-$\beta$1	Marenzana et al. (2006)
				$$\hbox {RMT} = 53\ \upmu \hbox {N}/10^6\,\hbox {cells}$$	
	456 (Max.)			15.0 ng/ml TGF-$\beta$1	
				$$\hbox {RMT} = 40\ \upmu \hbox {N}/10^6\,\hbox {cells}$$	
NIH 3T3 FB	255(Plat.)	0.20	1.50	Uniaxially constrained	Eichinger et al. (2020)
	213 (Plat.)	0.50	2.50	Uniaxially constrained	
	187 (Plat.)	0.50	1.50	Uniaxially constrained	
	135 (Plat.)	0.50	0.80	Uniaxially constrained	
	128 (Plat.)	1.00	1.50	Uniaxially constrained	
	138 (Plat.)	1.00	1.50	Biaxially constrained x-direction	
	142 (Plat.)			Biaxially constrained y-direction	
Porcine pulmonary arterial smooth muscle cell	759 (Plat.)	1.00	1.00	Inner 25% of artery, healthy	Hall et al. (2007)
	1119 (Plat.)			Outer 50% of artery, healthy	
	524 (Plat.)			Inner 25% of artery, hypoxic	
	228 (Plat.)			Outer 50% of artery, hypoxic	

Experiments were conducted in a uniaxial setup with medium containing 10% FBS unless indicated differently. Forces refer to experiments without (or before) any external strain applied. The RMT was obtained 12 h after addition of the actin cytoskeleton disrupting agent cytochalasin D after 24 h in culture. Maximal forces (Max.) and plateau forces (Plat.) are normalized by the number of cells used in the experiment. Analogously to the studies with dermal fibroblast, the possibly more suitable normalization (force/cross-sectional area)/(number of cells/gel volume) was not possible due to a lack of data, again suggesting a need to provide information about the cross-sectional area in future studies

### Biaxially constrained tissue equivalents

The inability of uniaxially constrained tissue equivalents to reproduce bi- or triaxial stress or strain states, which prevail in vivo, motivated the development of biaxial testing devices in the 2000s (Fig. 2d) (Knezevic et al. 2002; Thomopoulos et al. 2005, 2007; Humphrey et al. 2008; Sander et al. 2009; Hu et al. 2009, 2013; Lee et al. 2018; Eichinger et al. 2020). In many cases, however, biaxial settings have been used mainly for fairly general studies of fiber alignment and mechanical properties. Biaxial setups usually follow an approach very similar to the one of uniaxial ones. A pre-mixed matrix solution containing a prescribed number of cells is cast into a biaxial mold (mostly square-shaped or cruciform-shaped (Fig. 2c)) and is then allowed to set for some time. Insets are used on four sides so that weights or motors can be connected to the gel to apply external loading, or to constrain the gel in two directions to study cell-mediated compaction and the associated generation of tension. The following section briefly reviews key findings from biaxial experiments with tissue equivalents.

**Fiber alignment ** One drawback of uniaxial collagen gels compared to biaxial gels is that, due to the experimental setup itself, a strong structural and therefore mechanical anisotropy arises. This anisotropy is characterized by an alignment of collagen fibers and therefore a stiffening in the constrained direction. Interestingly, it was shown that the intensity of the anisotropy induced this way by the boundary conditions was the same for tendon and cardiac fibroblasts (Thomopoulos et al. 2005). Conversely, fibers in cruciform-shaped biaxial gels were found to be randomly oriented in the central region, suggesting an isotropic response, whereas in the arms, uniaxial conditions induced a fiber alignment parallel to the axis of the arms (Hu et al. 2009). Also, the influence of external loading on fiber orientation was studied. If cruciform gels seeded with dermal fibroblasts were loaded in only one direction, fibers in the gel appeared to align in this direction also in the central region of the gel. If these gels were subsequently unloaded and then loaded orthogonally to the first loading direction, cells were found to remodel the fibers in the central region of the gel such that they first became randomly distributed again. After prolonged loading, finally, an anisotropic fiber alignment in the new loading direction was observed (Lee et al. 2008).

**Mechanical properties ** The influence of static and cyclic equibiaxial stretching on the mechanical properties of fibroblast-seeded collagen gels has been studied over multiple days ( Chen et al. (2018)). Lee et al. (2018) observed that cyclic loading increased the stiffness more than static loading. Interestingly, the increased stiffness could be maintained even after cell lysis, again supporting the concept of inelastic cell-mediated matrix remodeling known already from uniaxial experiments (Marenzana et al. 2006). Based on second harmonic generation images, the enhancement of the mechanical properties was explained by thickening of collagen fibers in case of cyclic stretching (Lee et al. 2018).

**Mechanical homeostasis in higher dimensions** Regulation of matrix tension has only recently been studied in a fully controllable biaxial setup (Eichinger et al. 2020). In this study, NIH 3T3 fibroblasts were used (Fig 2c, d). Homeostatic tension in the biaxial setting was found to be higher and to be reached faster compared to a uniaxial setting (under otherwise identical conditions). Perturbations of the homeostatic state were studied under both equi-biaxial and strip-biaxial loading conditions. Notably, this was the first experiment to study relaxation after perturbation for up to $10\ \text{h}$ (compared to less than $60\ \text{min}$ in similar studies (Brown et al. 1998; Ezra et al. 2010)). This provided valuable information about whether the homeostatic state is fully or only partially restored after perturbations. When gels were released, gel tension was observed to increase above the prior level. By contrast, in case of a stretch perturbation, only a $\sim 5\%$ offset was observed to remain compared to the prior tension. So far, it is not yet clear how these observations can be understood and to which extent they reflect general properties of mechanical homeostasis and tissue equivalents, e.g., considering that NIH 3T3 fibroblasts were used.

In an even more recent study, the mechanosensitive response of collagen gels seeded with primary aortic smooth muscle cells to a step-wise stretch was studied (Eichinger et al. 2021a). Both for release and extension by $1\%$, which corresponded to a perturbation of $\sim 25\%$ of the homeostatic state force, the gel tension returned toward the prior step within a tolerance of $\sim10\%$.

## Discussion and conclusion

**Fig. 4:**
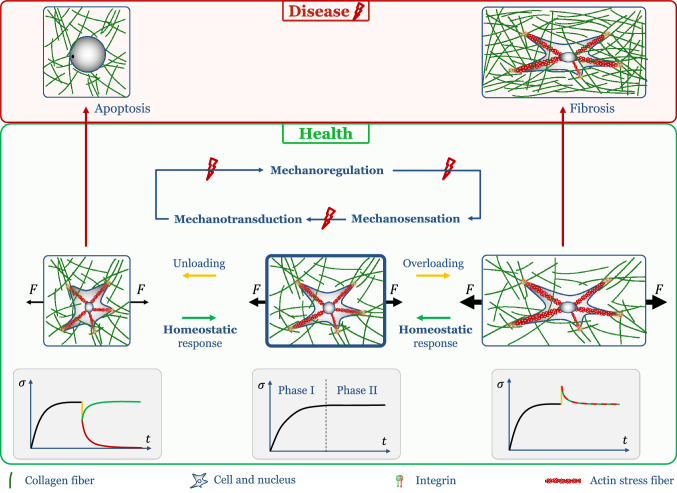
Schematic drawing of cell-matrix interactions in health and disease. Center: cell in normal conditions interacting via integrins with surrounding ECM, which in vivo typically exhibits some tension. Bottom row shows typical behavior of tissue equivalents developing a homeostatic nonzero plateau of tension over time. Right: In case of further increases in tension, i.e., overloading, healthy tissue seeks to restore the prior mechanical state due to a homeostatic feedback loop consisting of mechanosensation, mechanotransduction, and mechanoregulation. If this feedback loop is compromised, pathological signaling can lead to a fibrotic response (top right). In general, both the healthy and the fibrotic reaction may help to restore the preferred mechanical state (bottom right). Left: in case of decreases in tension, i.e., under-loading, e.g., due to injury, homeostatic feedback loops can lead to re-establishment of a homeostatic state. By contrast, pathological mechanosensitivity of tissues can lead to apoptosis (top row). The homeostatic feedback loop aims at restoring the homeostatic state, whereas apoptosis may lead to tissue failure (bottom row)

Studies with tissue equivalents have significantly increased our understanding of mechanobiology over the past few decades. Tissue equivalents represent controllable experimental model systems to study the evolution of biomechanical properties and mechanobiological responses of native tissues and tissue-engineered constructs with resident cells in a mechanically and chemically controlled three-dimensional matrix environment. Circular, cell-seeded free-floating collagen gels compact strongly over multiple days due to cell contraction (Simon et al. 2012, 2014). If similar gels are constrained in a uni- or biaxial setting at the boundaries and are therefore not able to deform the gel in the direction of loading, a two-stage response is generally observed: first, cells rapidly build up a certain level of tension (phase I), which is subsequently maintained (phase II) for a prolonged period (Brown et al. 1998; Jenkins et al. 1999; Brown et al. 2002; Sethi et al. 2002; Campbell et al. 2003; Marenzana et al. 2006; Karamichos et al. 2007; Dahlmann-Noor et al. 2007; Ezra et al. 2010; Courderot-masuyer 2017; Eichinger et al. 2020). The level of homeostatic tension appears to increase with both collagen concentration and cell density. By contrast, the rate of change leading to the homeostatic plateau tension seems to depend on the cell density but not on the collagen density. When the homeostatic state is perturbed mechanically, it appears that tissue equivalents work to re-establish the prior state within a particular range or tolerance. The exact response tends to depend, however, on the cell type and experimental conditions, including the presence of exogenous growth factors and ions that support different types of cellular activities, including matrix cross-linking (Brown et al. 1998; Ezra et al. 2010; Simon et al. 2014; Eichinger et al. 2020) (see Tables 1 and 2 for a collection of data from the literature and Fig. 4 for an overview).

Despite the impact of the aforementioned studies on our understanding of mechanobiology, many available experimental data are subject to at least one of the following three drawbacks (most drawbacks (i) and (ii)):

(i) Gels were subjected to uniaxial loading only, although most soft tissues are subjected to multiaxial mechanical states in vivo, with notable exceptions being tendons and some ligaments.
(ii) Relaxation intervals following external mechanical perturbations were often restricted to one hour or less, not allowing a definite answer within which tolerance the homeostatic state is restored after perturbations.
(iii) Gels were seeded with cells from an immortalized line; thus, they are of little biological interest due to genetic and phenotypic changes resulting from multiple years of culture.

Besides these limitations, one must remember that in vivo conditions are necessarily much more complex than either in vitro or ex vivo conditions. Cells reside in vivo within a complex extracellular matrix consisting of myriad proteins, glycoproteins, and glycosaminoglycans, subject to complex aperiodic mechanical loading and influenced by paracrine signaling from multiple cell types, including inflammatory. Nevertheless, much has been learned and much can yet be learned with simple model systems. In the following, we summarize some of the most pressing remaining questions.

**How does mechanical homeostasis arise?** Probably the most important question yet to be answered is: which mechanosensitive mechanisms and processes at the level of the cell (Weng et al. 2016) and of the tissue (Bhole et al. 2009; Flynn et al. 2010) together give rise to what we call mechanical homeostasis on the macroscale? One key need in this regard is to identify which mechanical quantity the individual cells can sense in their micro-environment and how their response is translated to the macroscopic organ level. On the microscopic level, where cells and tissue fibers appear as discrete objects, continuum-scale quantities such as stress or strain, or quantities derived from them, are not well-defined. Thus, on this scale, cells can probably only sense and react to (changes of) forces or displacements (Humphrey 2001). On the tissue and organ scale, this microscale behavior may give rise to an emergent behavior where continuum quantities such as stress or strain are effectively regulated. The micro-biomechanical and biochemical mechanisms of cellular mechano-sensing, transduction of cues into cellular regulative reactions via intracellular signaling pathways, and the controlled production, prestressing and degradation of extracellular matrix remain poorly understood. Tissue culture experiments are simple systems where accurately controlled stress and strain states can be imposed to study cellular responses. Especially interesting would be the use of cells with induced defects to study exact roles of specific components of the intracellular control system that gives rise to mechanical homeostasis. Similarly, cell treatment with drugs that just target one specific mechanism could provide important insights.

**Which regulatory processes govern mechanical homeostasis on short and long time scales?** In addition to different spatial scales, different time scales also need to be considered. Mechanical homeostasis is closely related to growth and remodeling. The latter refers to the inelastic reorganization of the microstructure of tissues, whereas growth describes the process of production and removal of tissue mass. This implies that mechanical homeostasis probably involves at least two very different biophysical processes, which can be expected to evolve in general at different time scales. Remodeling appears to regulate the mechanical state on the time scale of hours to days. Structurally significant mass turnover, that is, production and removal of tissue mass, most likely evolves over days to weeks (Nakagawa et al. 1989; Matsumoto and Hayashi 1994), and over such prolonged periods, mechanical homeostasis has not yet been studied using tissue equivalents given the challenges with long-term culture.

**What is the target of mechanical homeostasis? ** So far, it remains controversial which mechanical quantity might be the target of mechanical homeostasis. There is evidence that it may be closely linked to tension in particular tissues because a wide range of different animal species, differing in size and body weight over multiple orders of magnitude, develop nearly the same tension per medial lamellar unit (or wall stress) in the aorta (Wolinsky and Glagov 1967). Indeed, externally perturbed tissue equivalents appear to restore their tension toward the level prior to the perturbation (Delvoye et al. 1991; Brown et al. 1998; Ezra et al. 2010). Yet, other equally striking studies report that wall stiffness at mean blood pressure also tends to be nearly the same in the aorta across various invertebrates and vertebrates (Shadwick 1999). Könnig et al. (2018) yet reported that fibroblast-populated biomaterial scaffolds reacted differently when subjected to environments with different stiffnesses. In a 2-week period, compaction was significantly less and tension higher with stiffer surroundings. Other recent investigations suggest loading rate as a crucial parameter for mechanobiological homeostasis on the cellular scale (Zhu et al. 2016; Elosegui-Artola et al. 2018). This leaves open the key question which target quantity is governing mechanical homeostasis at the tissue and organ level: is it strain, tension/stress, stiffness, stress or strain rate, or some other more complex quantity? To answer this and other related questions, it could, for example, be helpful to develop tissue culture experiments where such quantities can be controlled independently for prolonged periods.

**What is the homeostatic range and when does homeostasis become adaptive?** Most in vitro studies of mechanical homeostasis in tissue equivalents are restricted to periods within which it is often hard to decide at which tolerance mechanical homeostasis aims to restore a certain target state. Moreover, it still remains unclear how and when the homeostatic target value or the tolerance around it change, following the concept of adaptive homeostasis (Davies 2016).

**What does mechanical homeostasis mean in higher dimensions?** In vivo observations so far focused on the component of tension in the circumferential direction of blood vessels (Wolinsky and Glagov 1967; Shadwick 1999), not allowing a generalization of the concept of mechanobiological homeostasis to higher dimensions. Similarly, in tissue cultures, quantitative studies of the homeostatic plateau tension have been performed almost exclusively in uniaxial settings. This is a major concern noting that in vivo, living tissues are predominantly subjected to complex multiaxial loading. To close the resulting gap in our understanding of mechanical homeostasis, there is a pressing need for more studies in multiaxial settings, with coordinate invariant mechanical metrics important to consider (e.g., although stress may be uniaxial with respect to the long-axis of a uniaxially loaded sample, shear stress yet exists relative to other coordinate systems, hence even in simple cases, we do not really know what component or collection of components of stress the cells may respond to). Additional studies and concepts will be needed to answer the question what mechanical homeostasis exactly means in higher dimensions.

**What are the different targets of different cell types and what is the influence of tissue origin?** So far, limited knowledge is available on how cell type and tissue origin influence mechanical homeostasis in tissue equivalents. In vivo, cells from different tissues have been exposed to different loading and different environments, which in general can be expected to affect cell function (Sewanan et al. 2019). A key question in this regard is how and for how long do cells remember their specific prior function when transferred to an artificial environment in vitro. Moreover, given that a basic tenet of experimentation is to keep everything the same except the one variable of interest, the question is how to quantify and assess the prior function and environment of cells used in tissue culture experiments. Alternatively, one may think about more complex ex vivo experimental devices and strategies, but matrix complexity remains complicated. In this regard, using matrices from decellularized tissues (as in tissue engineering) needs further attention.

As mentioned before, a major limitation of many available studies is the use of immortalized cell lines, or the frequent use of fibroblasts in cases of primary cells. The extension of these studies to other differentiated cells, stem cells (Butler et al. 2010; Leong et al. 2010), and cells stemming from diseased, for example, fibrotic or aneurysmal tissues, with possible defects that may, for example, affect cell-matrix interactions, will be helpful to further understand mechanical homeostasis in health and disease.

Importantly, to allow better comparisons across studies, more consistent experimental guidelines should be adopted. Such is difficult, however, given the many different methods, cell types, tissue types, species, ages, disease conditions, and so forth. At the minimum, there is a need for more precise reporting. It may be reported that vascular smooth muscle cells were harvested from the aorta, for example, yet these cells arise from different embryonic origins when contrasting the aortic root, ascending aorta, and abdominal aorta. Site-specific differences may be critical determinants of findings in culture. One can also read that dermal fibroblasts were used; from where on the body were these cells taken, however, and what was the age and sex of the donor are often undocumented pieces of information that may be critical in comparing results. For example, it was even shown that human ocular fibroblasts from different regions of the eye produce significantly different tension (Dahlmann-Noor et al. 2007). Of course, precise information on culture medium and gel constituents or gel preparation time should be given since they influence the behavior of cells with respect to mechanical homeostasis. Similarly, also cell passage number (Sawadkar et al. 2020) and isolation process (Eastwood et al. 1996) have a major impact. Finally, normalization of the measured data could prove useful, as, for example, a normalized force $F_n$,

1 $$\begin{aligned} F_n&= \frac{\text {force/cross-sectional\,area}}{\text {number\,of\,cells/gel\,volume}}, \end{aligned}$$

would allow a better comparison between different studies, although information about the dimensions of the tissue equivalents (cross-sectional area, length, volume) remains indispensable. By including this information in future reports, it may be possible to develop dimensionless quantities characterizing the mechanobiological properties of tissue equivalents, following the example of fluid mechanics where dimensionless quantities such as the Reynolds or Stokes number have greatly contributed to our understanding of complex physical systems.

Summing up, experiments with tissue equivalents have contributed significantly to our understanding of mechanobiology. Nevertheless, many key questions have not yet been addressed, or at least not fully. Mathematical modeling of mechanobiology is a fast growing field and could help tremendously in understanding the foundations of mechanical homeostasis (Holzapfel et al. 2000; Wakatsuki et al. 2000; Humphrey and Rajagopal 2002; Watton et al. 2004; Marquez et al. 2005; Mauri et al. 2016; Loerakker et al. 2016; Cyron et al. 2016; Braeu et al. 2017; Ban et al. 2019; Domaschke et al. 2019; Eichinger et al. 2021a, b). To support mathematical modeling, it will be particularly important to collect larger sets of reliable quantitative experimental data, especially about bi- or triaxial mechanical states. Understanding the exact mechanisms by which cells sense and regulate their mechanical microenvironment and how these mechanisms macroscopically affect tissues will be key for the development of therapies against numerous diseases such as aneurysms or cancer. Moreover, it will be of great importance for future regenerative medicine in general.

## Supplementary Information

Below is the link to the electronic supplementary material.

Supplementary material 1 (pdf 535 KB)
