# Supplementary material for: Mechanical homeostasis in tissue equivalents: a review
Source: Biomech Model Mechanobiol. 2021 Mar 8;20(3):833–50. doi: 10.1007/s10237-021-01433-9 (PMC8154823; doi:10.1007/s10237-021-01433-9)
Supplement: Supplementary file 1 — Supplementary material 1 (pdf 535 KB) [file 10237_2021_1433_MOESM1_ESM.pdf]

## Supplementary Material

To facilitate a comparison of data published in the literature, we herein order these data with respect to some key features. It is noteworthy that all publications discussed in the following, except for one, conducted their experiments using a culture force monitor (Eastwood et al. 1994). This device was developed based on the isometric tension apparatus built by Kolodney and Wysolmerski (1992) and was extended by an additional computer-based data collection system. The only exception in this regard is Eichinger et al. (2020), who developed a novel biaxial bioreactor.

To render the data in the literature easier to compare, we postprocessed it in several steps. First, all data points were interpolated such that all reported force values corresponded to the same times in any data set. This step was necessary to add two data sets and to ensure that the smoothing filter applied later works correctly. All data points were interpolated using a uniform time vector with a time step of 0.02 $h$ for sufficient accuracy.

Thereafter, all force values were converted into gross force responses, meaning that the force response actually measured in the experiments with the cell-seeded collagen gels. Some articles do not directly present this response but only a form of net response. The net response is the gross response minus the force response of a cell-free collagen gel of the otherwise same type. To retrieve the gross force, the response of the cell-free collagen gel as it was given in the respective paper was added again to the net force provided in the paper. This postprocessing step is crucial to make the data from different papers comparable. After obtaining the gross force of each data set, all force values were normalized to the number of cells in the tissue equivalent because it has been shown that the more cells a gel contains, the higher the resulting force.

Finally, a smoothing filter was applied to all data sets, which facilitates data analysis. We used local regression with weighted linear least squares with a 2nd degree polynomial model. Each new data point was computed under consideration of 15% of the respective total number of data points. This percentage was found to be ideal to obtain a curve that was sufficiently smooth while still representing the original data sufficiently accurately.

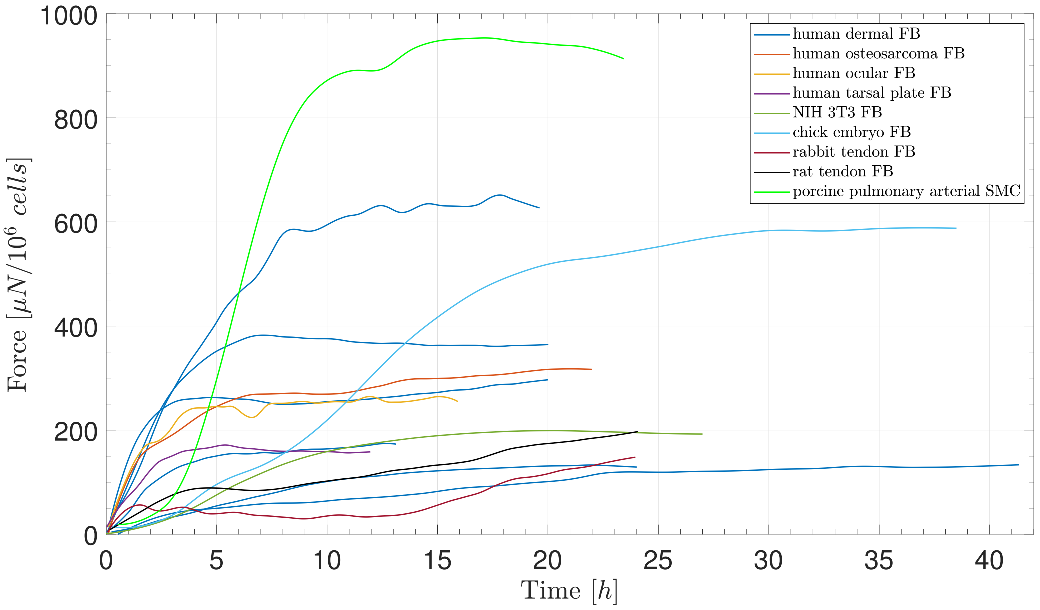


**Fig. S1** Each force curve/data point represents the results of a single scientific paper listed in Table 1 and 2. Uniaxial static experiments were considered studying healthy cells expressing $\alpha 2\beta 2$ integrins and using experimental medium containing 10% FBS without additional growth factors. If more than one data set fulfilling these criteria was published in a paper, the mean of the respective forces was computed. For human ocular fibroblasts (FB), only human Tenon's and human corneal fibroblasts were considered.

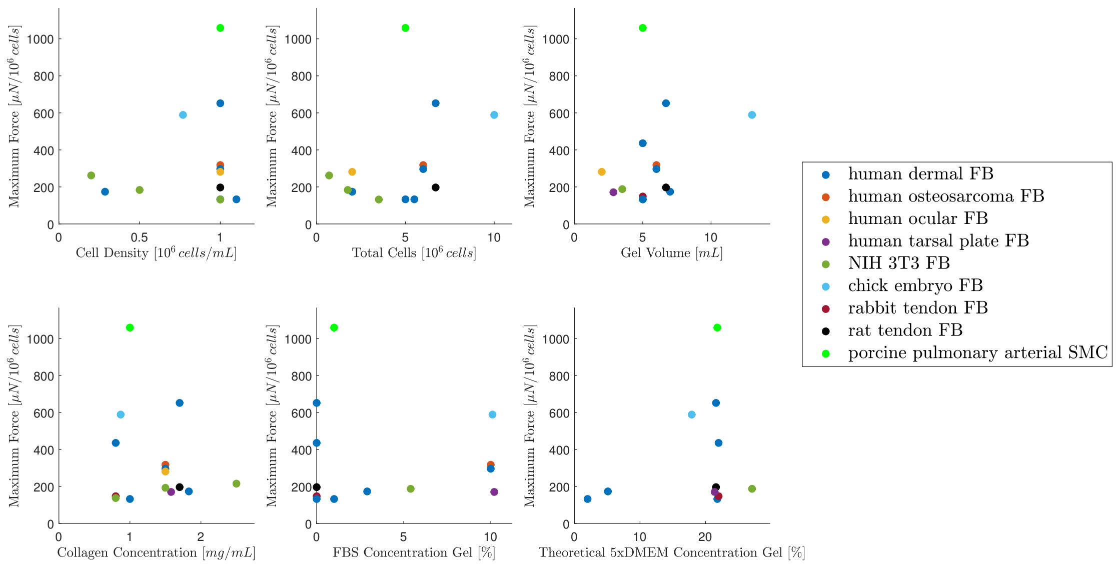


**Fig. S2** Point plot showing dependence of maximal force on cell density, total number of cells, gel volume, collagen concentration, FBS concentration and 5 x Dulbecco's modified Eagle's medium (DMEM) concentration

| **Gel Volume** [$mL$] | **Cell Type** | **Number of Cells** [$10^6$ $cells$] | **Collagen Concentr.** [$\frac{mg}{mL}$] | **FBS (Gel)** [$vol\%$] | **5xDMEM (Gel)** [$vol\%$] | **Other Gel Constituents** | **Reference** |
|---|---|---|---|---|---|---|---|
| 14.8 | human dermal FB<br>calf dermal FB | 3.0<br>6.0<br>3.0 | 0.46 | 10.0 | 18.0 | 19.32 mM HEPES,<br>3.05 mM NaOH | Delvoye et al. (1991) |
| 5.0 | human dermal FB | 5.5 | 1.00 | 1.0 | 21.8 | 0.1% glutamine,<br>0.1% Pen-Strep,<br>2.5 mM HEPES,<br>NaOH | Eastwood et al. (1994) |
| 5.0 | human dermal FB<br>rabbit tendon FB | unknown | 0.80 | 0.0 | 22.0 | NaOH | Eastwood et al. (1996) |
| 6.0 | human dermal FB<br>human osteosarcoma FB | 6.0 | 1.50 | 10.0 | unknown | NaOH | Jenkins et al. (1999) |
| 6.7 | human dermal FB | 6.7 | 1.70 | 0.0 | 21.6 | 15 µM HCl,<br>NaOH | Brown et al. (2002) |
| 7.0 | human dermal FB | 2.0 | 1.83 | 2.9 | 5.1 | 7.14% 10×PBS,<br>7.14 mM NaOH | Campbell et al. (2003) |
| 5.0 | human dermal FB | 5.0 | unknown | 0.0 | 2.0 | 10% 10×MEM,<br>NaOH | Karamichos et al. (2007) |
| 2.0 | human ocular FB | 14.0<br>scleral<br>2.0<br>Tenon's, corneal | 1.50 | unknown | unknown | L-glutamine,<br>$NaHCO_3$,<br>NaOH | Dahlmann-Noor et al. (2007) |
| 2.85 | human tarsal plate FB | 2.85 | 1.58 | 10.2 | 21.4 | 1.1% L-glutamine,<br>0.21% $NaHCO_3$ | Ezra et al. (2010) |
| 5.0 | human fascial tissue FB | 5.0 | 1.88 | 1.0 | 1.8 | 8.2% 10×MEM,<br>0.2 mM L-glutamine,<br>10 U/ml, 10 µg/ml Pen-Strep,<br>100 mM HEPES | Bisson et al. (2004)<br>Bisson et al. (2009) |
| 13.0 | chick embryo FB | 10.0 | 0.87 | 10.0 | 17.9 | Pen-Strep,<br>2.90 mM NaOH | Kolodney and Wysolmerski (1992) |
| 5.0 | rabbit tendon FB | 5.0 | unknown | 0.0 | 2.0 | 10% 10×MEM,<br>NaOH | Sawadkar et al. (2020) |
| 6.7 | rat tendon FB | 6.7 | 1.70 | 0.0 | 21.6 | HCl,<br>NaOH | Marenzana et al. (2006) |
| 3.5<br>uniaxial<br>6.5<br>biaxial | NIH 3T3 FB | 0.7<br>1.75<br>3.5<br>6.5 | 0.80<br>1.50<br>2.50<br>1.50 | 5.4 | 27.1 | 9.0 mM NaOH,<br>1.8 mM HEPES,<br>5.40% porcine serum,<br>0.54% Pen-Strep,<br>0.54% antibiotic/ antimycotic | Eichinger et al. (2020) |
| 5.0 | porcine pulmonary arterial SMC | 5.0 | 1.00 | 1.0 | 21.8 | 0.1% glutamine,<br>0.1% Pen-Strep,<br>2.5 mM HEPES,<br>NaOH | Hall et al. (2007) |

**Table S1** Supplementary information on gel constituents in previous publications. 5xDMEM concentration is a theoretical value representing the amount of 5xDMEM necessary to obtain the same final concentration of DMEM in the collagen gel as specified in the respective publication (although a differently concentrated DMEM might have been used).

| **Cell Type** | **Cell Density** $\left[\frac{10^6\,cells}{mL}\right]$ | **Isolation Process** | **Passage Number** | **Reference** |
|---|---|---|---|---|
| human dermal FB | 0.20<br>0.40 | explant growth | unknown | Delvoye et al. (1991) |
| human dermal FB | 1.10 | collagenase digestion,<br>explant growth | unknown | Eastwood et al. (1994) |
| human dermal FB | unknown | collagenase digestion,<br>explant growth (primarily) | unknown | Eastwood et al. (1996) |
| human dermal FB | 1.00 | explant growth | P6 - P9 | Jenkins et al. (1999) |
| human dermal FB | 1.00 | explant growth | P6 - P7 | Brown et al. (2002) |
| human dermal FB | 0.29 | unknown | unknown | Campbell et al. (2003) |
| human dermal FB | 1.00 | explant | P3 - P7 | Karamichos et al. (2007) |
| human osteosarcoma FB | 1.00 | unknown | unknown | Jenkins et al. (1999) |
| human ocular FB | 7.00<br>scleral<br>1.00<br>Tenon's,<br>corneal | dispase digestion | P2 - P10 | Dahlmann-Noor et al. (2007) |
| human tarsal plate FB | 1.00 | collagenase digestion | P3 - P4 | Ezra et al. (2010) |
| human fascial tissue FB | 1.00 | unknown | up to P5 | Bisson et al. (2004)<br>Bisson et al. (2009) |
| calf dermal FB | 0.20 | explant growth | unknown | Delvoye et al. (1991) |
| chick embryo FB | 0.77 | unknown | P2 | Kolodney and Wysolmerski (1992) |
| rabbit tendon FB | unknown | explant growth | unknown | Eastwood et al. (1996) |
| rabbit tendon FB | 1.00 | collagenase digestion | P0 - P6 | Sawadkar et al. (2020) |
| rat tendon FB | 1.00 | collagenase digestion,<br>explant growth | up to P8 | Marenzana et al. (2006) |
| NIH 3T3 FB | 0.20<br>0.50<br>1.00 | unknown | P4 - P5 | Eichinger et al. (2020) |
| porcine pulmonary arterial SMC | 1.00 | enzyme digestion | P3 - P6 | Hall et al. (2007) |

**Table S2** Supplementary information on cells used in previous publications.
